# Spectrum of external genital anomalies in disorders of Sex Development at Children Hospital & Institute of Child Health, Lahore, Pakistan

**DOI:** 10.12669/pjms.37.1.2991

**Published:** 2021

**Authors:** Sarah Khan, Raafea Tafweez, Areiba Haider, Muhammad Yaqoob

**Affiliations:** 1 Dr. Sarah Khan, MBBS, M-Phil Anatomy Assistant Professor of Anatomy, Department of Anatomy, King Edward Medical University, Lahore, Pakistan; 2 Dr. Raafea Tafweez, MBBS, M-Phil, FCPS, PhD. Professor of Anatomy, Department of Anatomy, King Edward Medical University, Lahore, Pakistan; 3 Dr. Areiba Haider, MBBS, M-Phil. Anatomy, Assistant Professor of Anatomy, Department of Anatomy, King Edward Medical University, Lahore, Pakistan; 4 Dr. Muhammad Yaqoob, MBBS, MCPS, PhD. Associate Professor of Genetics, Department of Genetics, The Children’s Hospital & Institute of Child Health, Lahore, Pakistan.

**Keywords:** Ambiguous genitalia, CAH, DSD, Third gender

## Abstract

**Objective:**

To describe the mode of presentation and frequency of external genital anomalies in disorder of sex development (DSD)

**Methods:**

This cross-sectional study was conducted at Children Hospital & Institute of Child Health, Lahore from January to December, 2016 on Children with DSD above 10 years of age. A detailed history and physical examination were done. Positive findings were recorded on a predesigned proforma and analyzed by SPSS 21. Karyotyping on blood samples was done to determine their genetic sex.

**Results:**

Out of 83 DSD children, 67% (n=56) were assigned a female sex at birth of which 9% (n=5) had ambiguous genitalia. Male sex at birth was given to 33% (n=27) of which 96% (n=26) had genital ambiguity. Mode of presentation other than ambiguous genitalia were delayed puberty, amenorrhea, hirsuitism, gynaecomastia, cyclic hematuria etc. Clitoromegaly was the main finding in 62.5% (n=5) and micropenis in 45% (n=9). Karyotypic sex of 56 female sex of rearing was 46XX 80% (n=45), 45X0 13% (n=7), XXX 2% (n=1) and 46 XY in 5% (n=3). Karyotypic sex of 27 male sex of rearing was 46XY in 78% (n=21), 46XX in 15% (n=4) and 47XXY in 7% (n=2).

**Conclusion:**

Disorders of sex development presented with a wide spectrum of external genital anomalies ranging from clitoromegaly in females to micropenis and hypospadias in males. There was also an extreme diversity in mode of presentation of these cases including pubertal delay, amenorrhea in females and gender confusion disorders.

## INTRODUCTION

The most fundamental aspect of early human development is the establishment of sex which is strictly controlled by a number of genes (SRY, SF1, SOX9 and others.^1^ Development of the reproductive system occurs as a result of sex determination and differentiation.^2^ Sex determination depends on the fact that whether an X bearing sperm or a Y bearing sperm fertilizes an oocyte.^2^

Disorders of sex development (DSD) are the congenital conditions in which the development of chromosomal, gonadal or anatomical sex is atypical.^3^ In 2006, Lawson Wilkins Paediatric Endocrine Society (LWPES) and European Society for Paediatric Endocrinology (ESPE) classified these disorders into three diagnostic categories: 46, XY DSD (formerly male pseudohermaphrodites), 46 XX DSD (formerly female pseudohermaphrodites) and sex chromosome DSD.^4^

Genital ambiguity is the most common presentation in DSD with a worldwide prevalence of about 1:4500.^5^ Congenital adrenal hyperplasia (CAH) is the most common cause of ambiguous genitalia in females with an incidence of 1 per 15000 live births in Great Britain.^6^ In Germany 2/10,000 cases were reported with ambiguous genitalia at birth.^7^

Poverty, lack of education, awareness, tendency of hiding genital ambiguity and social stigmatization are the main factors for late presentations in developing countries. 8 A detailed history, physical examination and early karyotypic analysis is playing a major role in diagnosis of appropriate sex before costly investigations.^9^ History should include drug intake during pregnancy, fertility, family history of genital anomalies, age of development of secondary sex characters, amenorrhea and consanguinity. 9

The evaluation of a phenotype of an apparent male with ambiguous genitalia is done by External Masculinization Score (EMS) ranging from 0–12 and a score less than seven is considered as genital ambiguity.^10^ A stretched penile length less than 2.5 standard deviations below the mean for age group is considered as micropenis.^11^

Congenital adrenal hyperplasia (CAH) is the most common cause of virilization in females mainly presenting with clitoromegaly and signs of androgen excess.^6^ Degree of virilization in an apparent female is determined by Prader’s scale.^12^This scale consists of five stages ranging from (stage I), a phenotypic female with mild clitoromegaly to (stage V) a phenotypic male with hypospadias.^12^

The aim of the study was to describe the mode of presentation and frequency of various forms of genital anomalies in DSD. This study will help medical professionals in recognizing genital ambiguity for assigning an appropriate sex as soon as possible before time taking and costly investigations.

## METHODS

This observational study was conducted on 83 children presented with any form of DSD at The Children Hospital & Institute of Child Health, Lahore from January to December, 2016 after taking ethical approval from the Institutional Review Board (IRB) (Ref No. 51/RC/KEMU, dated July 3, 2014). Children above10 years of age with any form of DSD were included in the study after taking an informed consent from their parents. Cases who have undergone any surgical correction procedure for genital ambiguity were excluded.

Parameters in history included age at the time of presentation, sex given at birth, consanguinity, fertility, maternal drug intake especially androgens, family history of genital ambiguity or other congenital anomalies, age at menarche, amenorrhea, hirsuitism, delayed puberty, cyclic hematuria etc.

Physical examination was done in a separate room with comfortable environment in the presence of parents and under the supervision of a trained specialist. Complete privacy and confidentiality of all the cases were maintained. Blood pressure, height and weight were measured. The primary concern on genital examination was to find out the presence of palpable gonads in scrotal sacs or labial folds. Females with DSD were examined for hirsuitism, breast development, clitoromegaly, posterior labial fusion, urethral and vaginal opening. Males with DSD were examined for the size of phallus, hypospadias, presence or absence of gonads, gynaecomastia and urogenital sinus. A ruler was used to measure the size of stretched phallus on dorsal aspect from pubic symphysis up to glans penis and a stretched penile length (SPL) less than 2.5 standard deviations below the mean for age was considered as micropenis. All the physical parameters were recorded on a predesigned proforma in the light of Tanner’s and Prader’s scoring system. Tanner scoring system was used to determine the pubertal age while Prader scoring was used to determine the degree of virilization of external genitalia. Only those cases were photographed who gave consent without any pressure or persuasion.

Karyotyping of all cases was done for the confirmation of genetic sex. For karyotyping 2ml of blood sample was collected in Lithium-Heparin vial. The heparinized blood sample was processed and the mononuclear cells were purified and cultured in a nutrient-enriched medium for a period of 72 hours at 37 degree Celsius. It was treated with colcemid to arrest the cells in metaphase of mitosis. Afterwards slides were prepared and stained with GIEMSA dye. The stained slides were analyzed under a compound microscope with a built in camera. A picture of chromosomes in spread form was shown on the computer screen. Chromosomes were arranged in pairs by their size, length of chromosome and placement of centromere with the help of a software called MAC TYPE 4. The chromosomes pairs were numbered from largest (number 1) to smallest (number 22). There were 22 pairs of chromosomes called autosomes which match up exactly and only one pair of the sex chromosomes in XX or XY combination were identified. Any deficient or extra copy of sex chromosome was specially looked for.

Data collected was entered and analyzed by using Statistical Package for Social Sciences (SPSS) Version 21 and presented in the form of frequencies and percentages.

## RESULTS

The study was conducted on 83 DSD children. The mean age at presentation was 14.5±1.5 years. A female sex at birth was given to 67% (n=56) and a male sex at birth was given to 33% (n=27) by their parents.

Genital ambiguity was the presenting complaint in 23 cases out of which 66% (n=18) had a male sex of rearing and 9% (n=5) had a female sex of rearing. Other complaints in cases with male sex of rearing were delayed puberty 30% (n=8), gynaecomastia 4% (n=1) and in female sex of rearing were primary amenorrhea in 62% (n=35), delayed puberty 25% (n=14) and hirsutism 4% (n=2).

**Fig.1:**
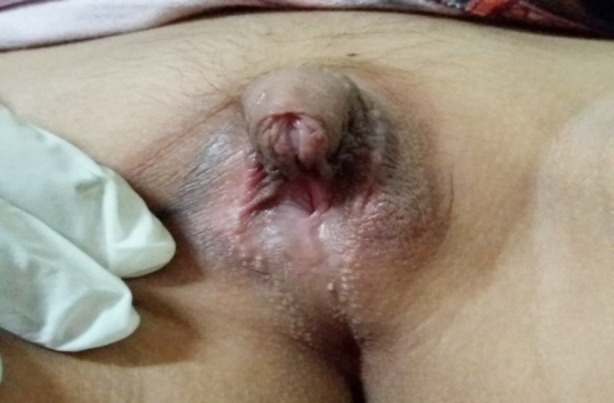
Clitoromegaly in a “14 -years old” female sex of rearing with 46XX karyotype.

**Fig.2:**
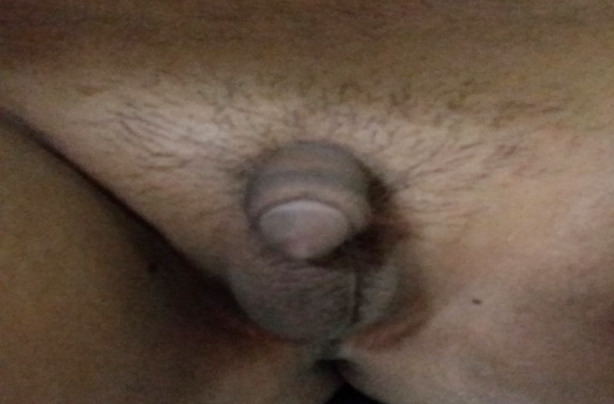
Micropenis and left undescended testis with 46XY karyotype.

On physical examination, we found that 5 more cases had ambiguous genitalia. making a total of 28 cases of genital ambiguity. Out of these five cases three were having a female sex of rearing and two with male sex of rearing. Isolated clitoromegaly was the main finding in 62.5% (n=5) females (Table-I, Fig.1) while micropenis was the main finding in 45% (n=9) in male sex of rearing. (Table-II, Fig.2).

**Table-I:** Physical examination of ambiguous genitalia in females.

Ambiguous genitals in females’ sex of rearing	n	%
Isolated clitoromegaly	5	62.5
Clitoromegaly with posterior labial fusion	1	12.5
Clitoromegaly with gonads in labial folds.	1	12.5
Bifid labioscrotal folds and absent vagina.	1	12.5

Total	8	100

**Table-II:** Physical examination of ambiguous genitalia in males.

Ambiguous genitals in males’ sex of rearing	n	%
Micropenis	9	45
Undescended testes	4	20
Hypospadias	3	15
Urogenital sinus and micropenis and hypospadias	2	10
Duplication of penis with penoscrotal hypospadias	1	5
Bifid scrotum with micropenis and hypospadias	1	5

Total	20	100

**Fig.3:**
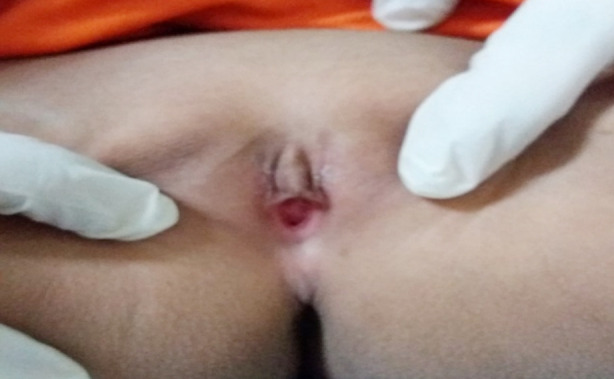
A case who was phenotypically female and genotypically male (46XY)

**Fig.4:**
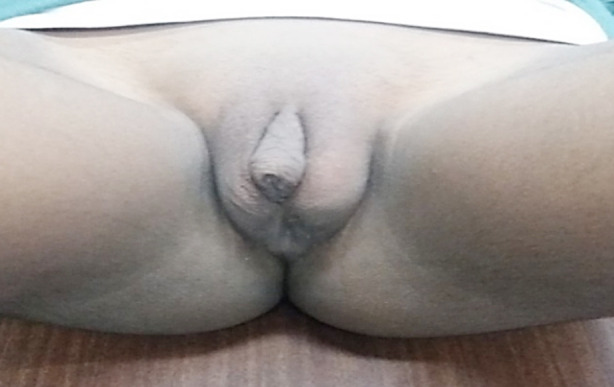
A case with clitoromegaly, urogenital sinus and bifid labioscrotal folds with 46XX karyotype.

Karyotypic analysis was done in all cases to have an idea about their genetic sex. (Table-III). There was an interesting case of female sex of rearing having ambiguous genitalia with a male karyotype 46, XY (Fig.3). One male sex of rearing had 46, XX karyotype. On genital examination clitoromegaly, bifid labioscrotal folds and urogenital sinus was observed (Fig.4).

**Table-III:** Karyotypic Analysis.

Karyotypic analysis	Number of cases(n)	Frequency (%)
Male 46XY	26	31.3
female 46XX	47	56.6
Turner’s syndrome 45 X0	7	8.4
Klinefelter’s syndrome 47 XXY	2	2.4
XXX females	1	1.2

Total	83	100

**Fig.5:**
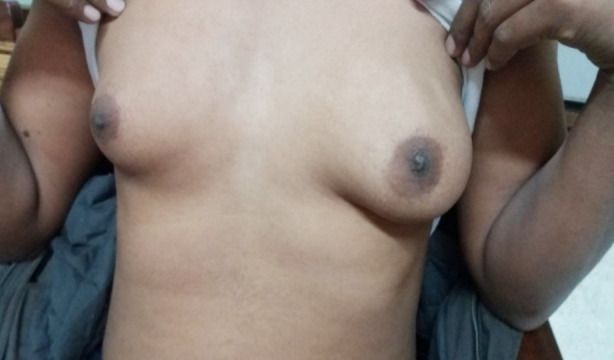
Bilateral gynaecomastia in male with 47XXY (Klinefelter’s syndrome).

**Fig.6:**
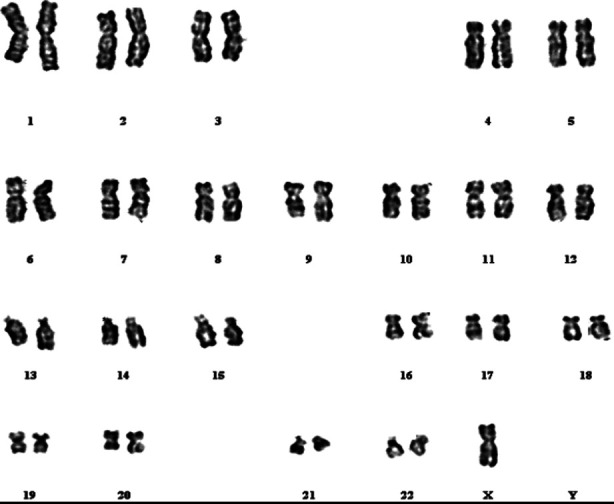
Karyogram of Turner’s Syndrome.

**Fig.7:**
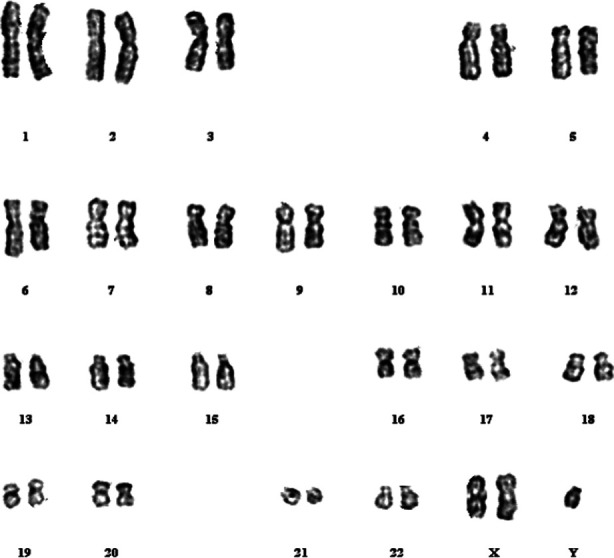
Karyogram of Klinefelter’s Syndrome.

Klinefelter’s syndrome was diagnosed on karyotyping in 2 (2.4%). (Table-III, Fig.7) Both of them had micropenis while one of them had marked gynaecomastia (Fig.5) Seven cases had Turner’s syndrome on karyotype (Table-III, Fig. 6) and were rightly assigned female sex at birth.

## DISCUSSION

In many parts of the world including our region the birth of an individual with genital ambiguity is considered as a social stigma.^13^ Genital ambiguity is a complicated inherited disorder with multiple presentations. Ambiguous genitalia pose a medical emergency because of physical, social and psychological factors.^13^ A correct diagnosis of DSD required a detailed clinical evaluation, hormonal and radiological analysis along with chromosomal and molecular evaluation.^14^

Age at presentation has a crucial role in the management of DSD. In this study we included DSD children above 10 years of age who did not get treatment at an early age or developed signs of DSD at a later age. The mean age at presentation was 14.5±1.5 years. The presenting age in other developing countries was reported as late adolescence or early adulthood.^15^ Similar data was recorded from a Turkey and India.^16^,^17^ On the other hand in developed countries sexual ambiguity is not a taboo because public awareness was given on the importance of early management by multiple societies and support groups. 15,18

Genital ambiguity was the major presenting complaint in 23 cases with 66% (n=18) male sex of rearing and 9% (n=5) female sex of rearing. This finding was consistent with studies in Pakistan^19^ and Turkey.^16^ It was interesting that on physical examination 5 more cases 33% (n=28) had ambiguous genitalia. In our study isolated clitoromegaly was the major finding in 62.5% (n=5) similar to studies in Egypt and Saudia Arabia with a frequency of 76.9% and 92.9% respectively. 20,21. Males with genital ambiguity showed micropenis at the top in 45% (n=09) leading to a variety of features such as undescended testes, scrotal anomalies, urogenital sinus, hypospadias. These findings were comparable with the findings of Nicolino.^22^ Micropenis was also a major finding in 25% of males with ambiguous genitalia in another study in Pakistan.^19^

The Chicago Consensus Conference classified isolated hypospadias among 46XY disorders of sexual differentiation.^04^ In present study the frequency of hypospadias was 15% comparable to another study in Karachi, Pakistan with the frequency of hypospadias as 12.5%.^19^ In Italy an overall hypospadias prevalence rate was 3.066 ± 0.99 per 1000 live births.^23^ The frequency of undescended testes was found to be 20% in this study. A population-based study in Bulgaria showed the prevalence of cryptorchidism as 1.52 % in boys up to 19 years.^24^

Turner’s syndrome and Klinefelter’s syndrome are categorized as disorders of sex chromosomes.^04^ Klinefelter’s syndrome is a consequence of an extra X-chromosome in males (46XXY) and is the commonest sex chromosomal disorder in one out of 600 males.^25^ The diagnosis of Klinefelter’s syndrome is rarely made before puberty because of paucity of clinical manifestations and normal male external genitalia at birth.^25^ Turner’s syndrome appears as a result of one missing X chromosome in females (45X0) and is diagnosed in late childhood or adolescence during investigation of short stature or delayed puberty.^26^ In this study the frequency of Turner’s syndrome 5% (n=7) was higher than Klinefelter’s syndrome 2.4% (n=2). All cases of Turner’s syndrome were given female sex of rearing at birth and their physical examination showed no sexual ambiguity. Higher frequency of Turner’s syndrome as compared to Klinefelter’s syndrome was also given by studies in Egypt,^27^ Turkey^16^ and Pakistan.^19^

In Pakistan, few studies have been done on DSD which are limited to review,^28^ case and data presentations.^29^,^30^ The present study explored the spectrum of genital anomalies in a tertiary care Hospital of Lahore, Pakistan. Because of the scanty data available on this topic in our country, there is a dire need to explore these disorders from different angles for proper management. The present study is a step towards future studies on this topic.

We recommend that the facilities of chromosomal analysis and SRY gene molecular analysis should be available in each tertiary care hospital and should be done as a routine part of the investigations. Furthermore prenatal DNA testing should be done in all high-risk families. There is a need for creating an awareness in general population through electronic, print and social media about DSD and their management.

## CONCLUSION

Disorders of sex development presented with a wide spectrum of external genital anomalies ranging from clitoromegaly in females to micropenis and hypospadias in males. There was also an extreme diversity in mode of presentation of these cases including pubertal delay, amenorrhea in females and gender confusion disorders.

### Author`s Contribution:

**SK:** Designed, data collection, Karyotyping, statistical analysis & manuscript writing.

**RT:** Conceived and designed the research, suggestions, proof reading & final approval.

**AH:** Statistical analysis & writing.

**MY:** Assist in Karyotyping & Proof reading.
